# In-hospital rotavirus vaccination in premature and medically ill infants: a systematic review of uptake and safety

**DOI:** 10.1186/s40348-026-00238-z

**Published:** 2026-05-12

**Authors:** Janna-Lina Kerth, Calvin Kurz, Jonas Obitz, Tim Vogel, Ertan Mayatepek, Mark Dzietko

**Affiliations:** 1 Department of General Pediatrics, Neonatology, and Pediatric Cardiology, University Hospital Düsseldorf, Heinrich-Heine-University, Moorenstr. 5, Düsseldorf, 40227 Germany; 2 Children’s Hospital, HELIOS Hospital Uelzen, Uelzen, Germany; 3 Praxis Kindergesundheit Torgau, Eilenburger Str. 26, 04860 Torgau, Germany

**Keywords:** Rotavirus Vaccines, Infant, Premature, Intensive Care Units, Neonatal, Immunization, Schedule, Vaccine Safety

## Abstract

**Background:**

Rotavirus vaccination is widely recommended in infancy and has substantially reduced morbidity from severe gastroenteritis. In premature and medically ill infants, prolonged hospitalization often interferes with timely vaccination. Despite recommendations to vaccinate clinically stable hospitalized infants according to chronological age, concerns regarding adverse events, apnea-bradycardia episodes, and nosocomial transmission continue to limit uptake in neonatal intensive care units (NICUs). Delayed vaccination may result in missed opportunities, as initiation after 24 weeks of age is contraindicated. The objective of this review was to systematically assess the evidence on the administration of rotavirus vaccines to premature and medically ill infants during their in-hospital stay (e.g., neonatal intensive care units or postnatal wards), and to evaluate the occurrence, type, and frequency of adverse events following such vaccination.

**Methods:**

This systematic review was conducted in accordance with PRISMA guidelines. Medline, Embase, and Web of Science were searched without restrictions on publication year or language. Eligible studies included premature or medically ill infants who received rotavirus vaccination during hospitalization. Randomized controlled trials, cohort studies, case–control studies, and case series with at least five infants were considered. Data were extracted on vaccination uptake, adverse events, viral shedding, and nosocomial infection. Risk of bias was assessed using Joanna Briggs Institute critical appraisal tools, and certainty of evidence was evaluated using the GRADE approach.

**Results:**

Fifteen observational studies including a total number of 5,443 infants met inclusion criteria; no randomized controlled trials were identified. Vaccination uptake varied widely and was generally low in routine clinical practice. Reported adverse events were predominantly mild and transient, with gastrointestinal symptoms being most common. Fever occurred in up to 10% of vaccinated infants. No cases of intussusception or volvulus were reported. Apnea-bradycardia episodes were inconsistently documented; where assessed, their frequency was comparable to baseline rates or to those observed after routine two-month vaccinations. Viral shedding of vaccine-strain rotavirus was frequently detected, particularly after the first dose; however, nosocomial transmission to unvaccinated infants was rare and not associated with symptomatic gastroenteritis. Overall certainty of evidence was low due to heterogeneity and moderate to high risk of bias.

**Conclusion:**

Available evidence suggests that in-hospital rotavirus vaccination in clinically stable premature and medically ill infants is generally safe and not associated with relevant nosocomial infection. Nevertheless, uptake remains inconsistent. Standardized protocols, provider education, and implementation-focused strategies are needed to reduce missed vaccination opportunities and ensure timely protection in this high-risk population.

**Systematic review registration (PROSPERO):**

CRD420251163827

**Supplementary Information:**

The online version contains supplementary material available at 10.1186/s40348-026-00238-z.

## Introduction

Rotavirus vaccination is widely recommended for infants worldwide [1–3] and has significantly reduced the global burden of severe gastroenteritis in early childhood [4]. While many national immunization programs advise administering the vaccine to clinically stable preterm or hospitalized infants according to their chronological age [5, 6], some countries—such as the United States [7] or Israel [8]—explicitly recommend delaying vaccination until hospital discharge. In practice, however, adherence to these guidelines remains inconsistent [9, 10]. Even in settings where in-hospital administration is endorsed, vaccination is frequently deferred, and many preterm infants surpass the upper age limit for the first dose [11]. Because rotavirus vaccination should not be initiated after 24 weeks of chronological age due to an increased risk of intussusception, these delays result in missed opportunities for protection among preterm or medically ill infants that have a higher risk for severe acute gastroenteritis [12, 13].

A rapid review by Sicard et al. in 2020 found no evidence to support withholding rotavirus vaccination in preterm or hospitalized infants on safety grounds [10]. Despite this, uncertainty and variability in clinical practice still persist. Therefore, this systematic review aims to synthesize evidence on in-hospital rotavirus vaccination, with a particular focus on severe adverse events and nosocomial infections. It further seeks to identify potential barriers to timely administration and to evaluate whether clinical practice has evolved in recent years in response to emerging evidence on vaccination safety.

## Methods

### Design

This systematic review followed the *Preferred Reporting Items for Systematic Reviews and Meta-Analyses* (PRISMA) guidelines [14]. It was registered with the International Prospective Register of Systematic Reviews (PROSPERO; registration ID: CRD420251163827).

## Search strategy

Three electronic databases (Medline, Embase, and Web of Science) were searched, complemented by the search of personal archives and manual screening of reference lists. The searches were conducted on 08 October 2025. There were no restrictions regarding year of publication and no restriction for language other than using English search terms (see Additional File 1).

### Study selection

#### Inclusion criteria

Studies were included if they involved premature or medically ill (i.e., in need of in-hospital treatment after birth) infants who received a rotavirus vaccine during their in-hospital stay, such as in neonatal intensive or intermediate care units. Eligible study designs included randomized controlled trials, prospective and retrospective cohort studies, case–control studies, and case series with at least five infants. Studies were required to report data on vaccination practices, uptake rates, feasibility, or adverse events following in-hospital administration of a rotavirus vaccine. No restrictions were applied regarding publication year, language, or geographical region.

#### Exclusion criteria

Studies were excluded if they focused exclusively on term, healthy infants or if rotavirus vaccination was administered only after hospital discharge. Publications without clear information on the timing of vaccination, animal or laboratory studies, case reports including fewer than five neonates, reviews, book chapters, commentaries, and editorials were excluded.

### Screening process

Records were imported into EndNote X9 (Clarivate Analytics, Philadelphia, PA, USA). A piloting of the screening was conducted jointly by two researchers for ten records for clarification and specification of inclusion and exclusion criteria. If information available in the published paper was not sufficient for making a judgment, authors were contacted for further information. If no response was received, articles were excluded from the analysis.

After removal of duplicates, titles and abstracts were screened by two researchers individually. If the available information did not suffice or the decision was not unanimous, the full text was assessed. Full texts were assessed for eligibility, and any disagreement between the researchers was resolved through discussion with a third researcher.

### Data extraction

Data were extracted for year of publication, journal, country of study, language of publication, study design, population and setting, number/rate of vaccinated infants, number/rate and type of adverse events, shedding, nosocomial infection number/rate, type of vaccine used and reason for non-vaccination. A short summary was included. If sufficiently detailed data were not available from the publication, authors were contacted for further information. Piloting included five full texts and led to refinements of categories and documentation requirements. Data extraction was carried out independently by two researchers.

### Risk of bias and quality of evidence assessment

Risk of bias was assessed using the Joanna Briggs Institute Checklists for Critical Appraisal [15].

Bias was assessed individually by two researchers after piloting. Quality of evidence was assessed according to GRADE guidelines for systematic reviews [16]. Discrepancies were resolved through debate without the need to involve another researcher. No inclusion or exclusion decisions were to be based on this assessment.

### Data synthesis

Data were synthesized quantitatively when reasonable and meaningful. If pooling of data was not feasible nor adequate, we chose a narrative, qualitative approach to summarize findings.

## Results

### Characteristics of included studies

**Fig. 1:**
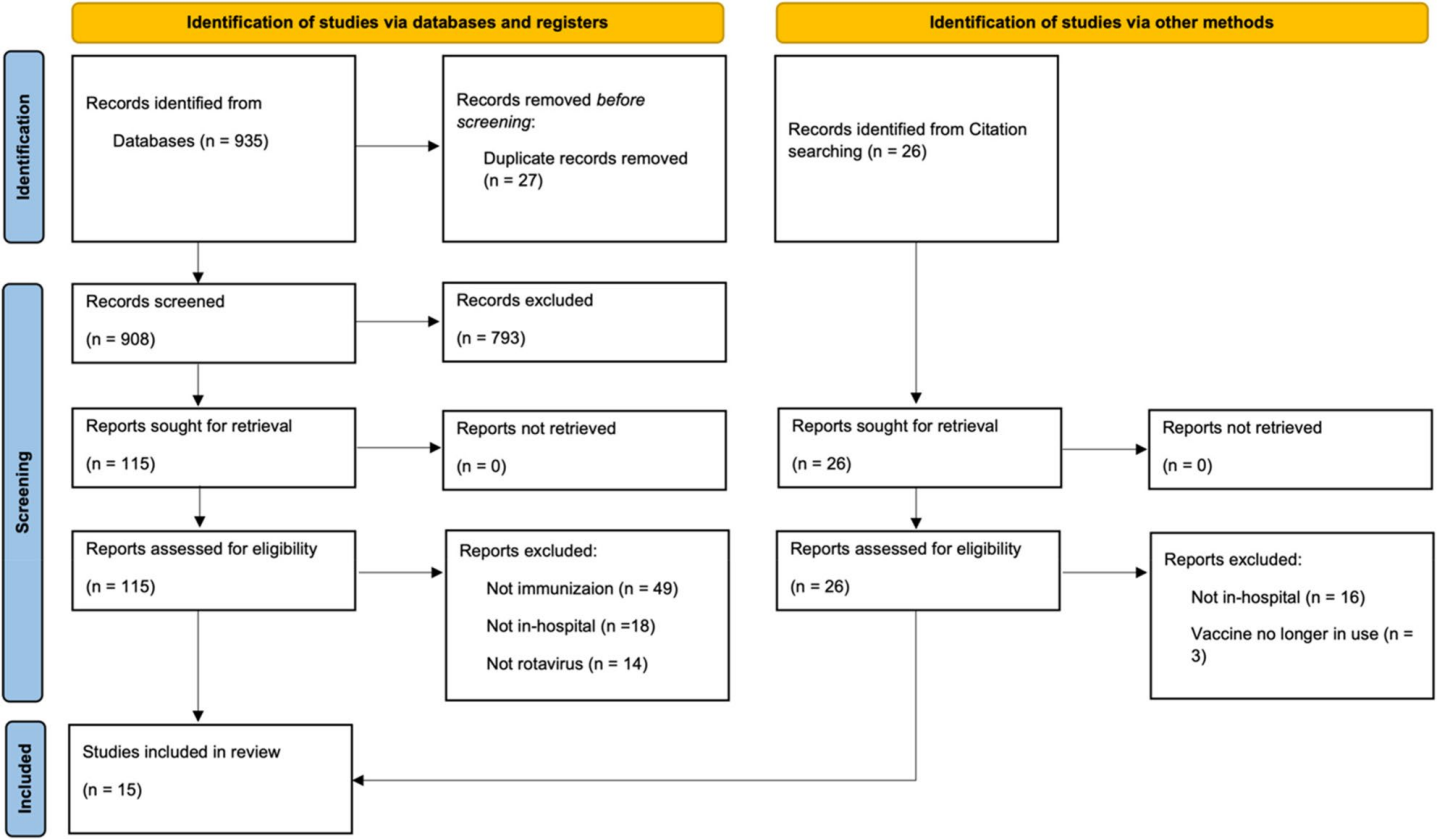
PRISMA flow chart. Source: Page MJ, et al. BMJ 2021;372:n71. doi: 10.1136/bmj.n71

After full text screening, 15 studies with a total number of 5,443 infants were included into the final analysis (see Fig. 1, PRISMA flow chart) [17–31]. The number of included infants per study varied greatly, with the smallest including only 9 and the largest including 1,991 infants. Not all studies explicitly reported the share of premature infants, but it was at least 78% (4,181/5,443) (see Supplementary Material: Table of Included Studies). Likewise, not all studies reported the number, but at least 460 patients with pre-existent gastrointestinal pathologies were included. However, results for this subgroup were not reported separately.

**Fig. 2:**
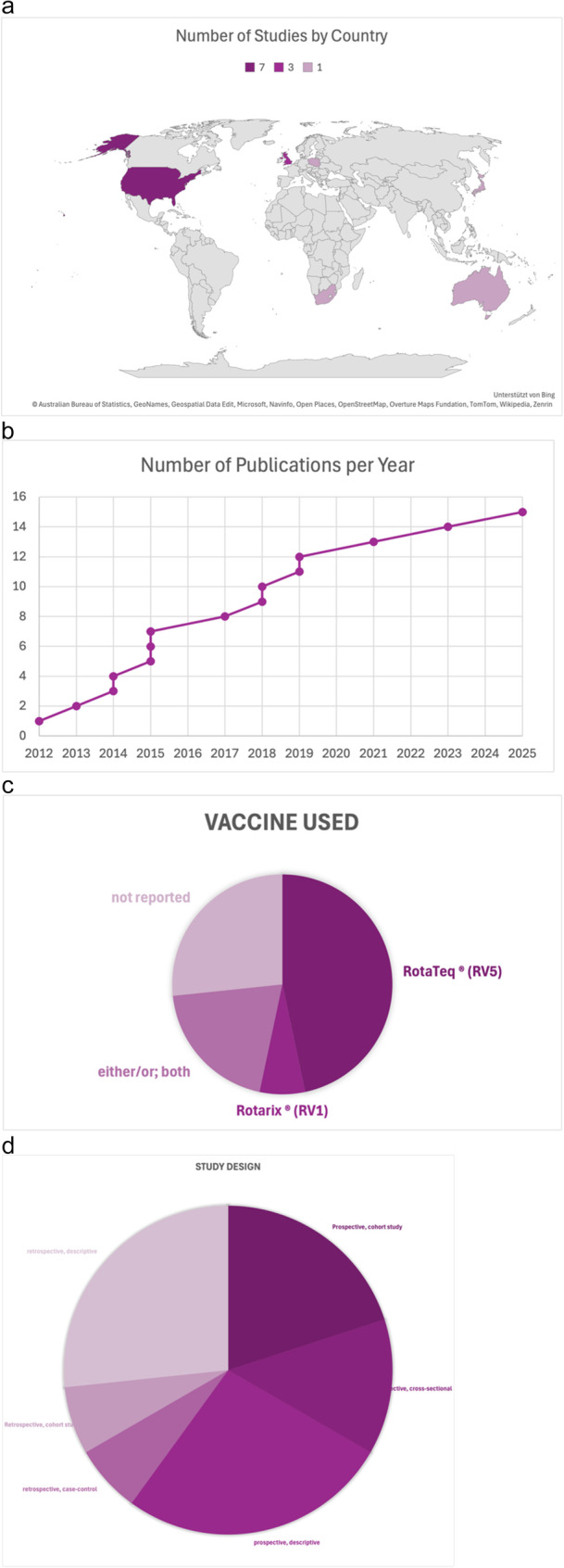
**a**-**d**: characteristics of included studies. **A** shows the number of publications per country; **B** shows the number of publications per year; **C** shows the type of vaccine used in the included studies; and **D** shows the study designs of included studies

Language of publication was English in all cases. No randomized controlled trials met our inclusion criteria and included studies were highly heterogenous regarding design, setting, sample, data collection and analysis. Characteristics of included studies can be found in Fig. 2.

### Risk of bias and quality of evidence

**Fig. 3:**
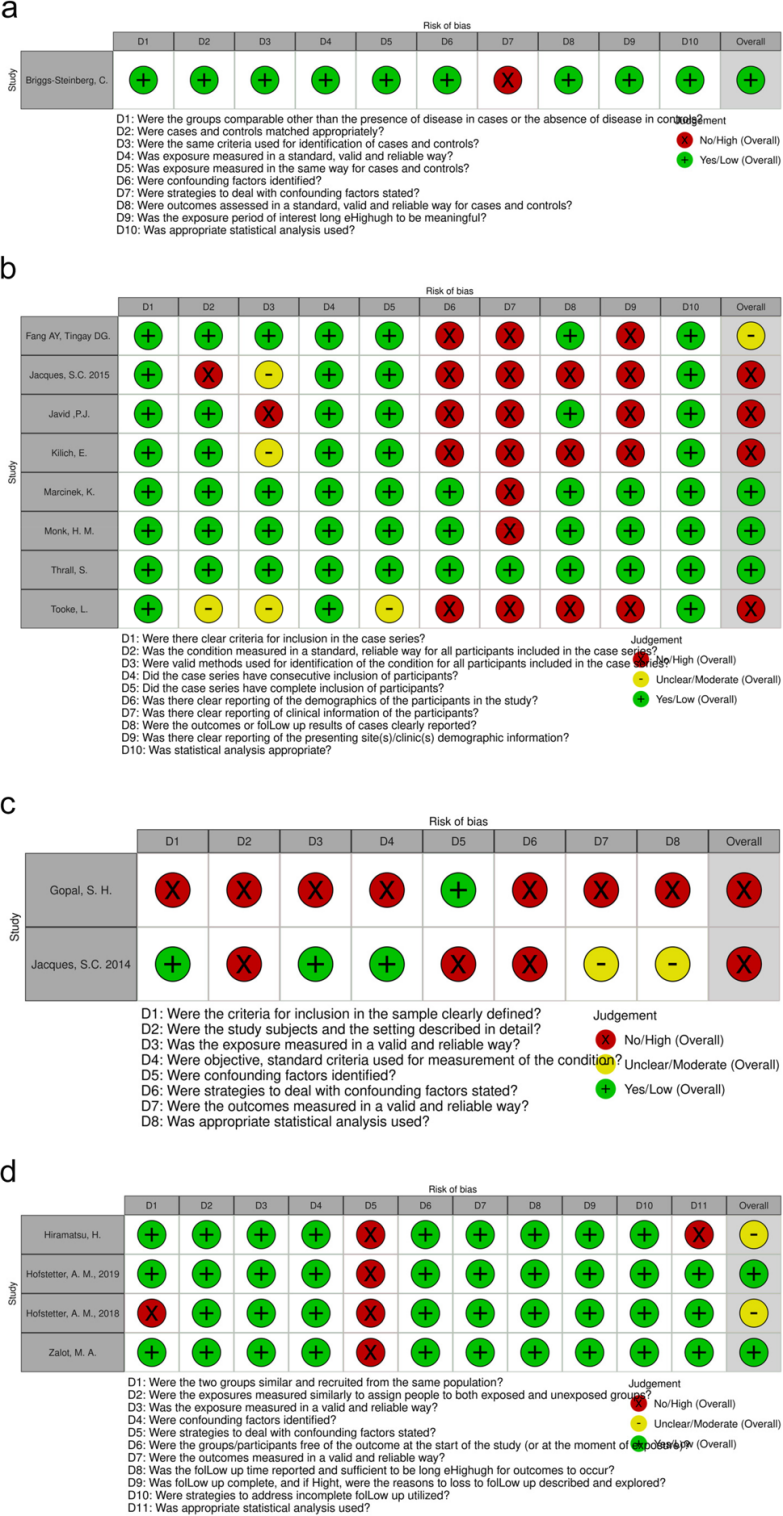
**a**-**d** show the risk of bias assessment for the included case control study, descriptive/case series studies, cross sectional studies, and cohort studies, respectively

We considered the quality of the existing evidence low for all available outcomes. Risk of bias was moderate to high for many studies (see Fig. 3). The inherently high variability of clinical presentation of hospitalized premature infants was identified as an important confounding factor for all studies, although some implemented measures to address this such as comparing events both pre- and post-vaccination in the same cohort as well as the vaccinated cohort to an unvaccinated historic control in one study [17]. There were no randomized controlled trials, and all evidence stems from observational studies, of which 6 were retrospective. Furthermore, there were few studies contributing to every outcome, often with a small number of patients and/or a low number of events recorded. The documentation of events was heterogenous, some only recording 72 h and others up to four weeks post-vaccination, partly relying on reviewing chart documentation. Different inclusion criteria, type of vaccination used, allowance of co-administration of other vaccinations, and setting further contributed to inconsistency. Lastly, the reported frequencies of events were very heterogenous for all outcomes other than nosocomial infections.

### Vaccine uptake

Vaccination rates reported differed highly between studies, partly due to study design. In those studies where study design did not necessarily lead to a change or only vaccinated children were included, vaccination rates were generally low. One retrospective analysis from the United States found that if hospitalization lasted longer than 104 days, only 3.5% of infants had received rotavirus vaccination (RV) [21]. In a study by the same group describing a program for routine administration of RV, however, still only 33% of age-eligible infants were up to date on their RV (compared to 83% for other vaccinations) and 43% had missed their time frame at the time of discharge [22]. In a study from Poland, vaccination rate increased after it was deemed refundable due to official recommendations and, according to the authors, possibly due to increased willingness to vaccinate during the Covid-19 pandemic (from 14% in 2019 to 73% in 2021) [25]. In a survey of US neonatologists, 93% stated that they did not routinely administer RV [19], in another survey of UK neonatal units (response rate 56/61, 92%), 45/56 NICUs reported vaccinating against rotavirus. Of those, only 37.7% (17/45) had a clinical practice guideline and 17/45 individually decided on a case-by-case basis [31].

Reasons for non-vaccination are listed in Table 1.

**Table 1:** Reasons for non-vaccination as reported in included studies

Lack of parental consent/parental request [19, 25]	
Medical reasons	Gastroenterological symptoms (large inguinal hernias; severe abdominal pain) [25]
	recurrent infections [25]
	Clinically unstable [19, 21, 28, 30]
	Low birth weight, low gestational age* [19]
	Surgical diagnoses/awaiting surgery [30, 31]
	Neutropenia [31]
Doubt of vaccine safety* [21]	
Communication issues* [21]	
Provider preference* [19]	

* reported for all vaccinations, not specifically for RV

### Type of vaccine used

Seven studies with a total of 670 vaccinated infants report having used the pentavalent rotavirus vaccination (RotaTeq ^®^, Merck & Co., Inc., 2023) [17, 18, 22, 26–29], one study with a total of 13 vaccinated infants used the monovalent rotavirus vaccination (Rotarix ^®^, GlaxoSmithKline Biologicals, 2023) [24], and three included children having received either [20, 23, 25]. The other studies did not report type of vaccination used.

### Adverse events

Three studies reported that no adverse events were recorded but did not provide more specific information [25, 28, 30].

In a retrospective study that reviewed case notes, clinical changes were noted in 23/96 (23.9%) infants after vaccination [26]. In the same period, 10/801 (1.2%) unvaccinated infants showed clinical changes. However, none of the symptoms were attributed to RV in their analysis. In a further retrospective study, alternative explanations for symptoms after RV were found in 69% of cases after chart review [27]. A study conducted in the United States in 2021 did not find an increase in diagnostic tests (i.e., abdominal x-rays, evaluation for sepsis) ordered after observance of clinical symptoms after RV [17].

An overview of adverse events is provided in Table 2.

**Table 2:** Adverse events reported in included studies

Adverse Event	Number of Studies Reporting the Outcome	Total number of vaccinated infants	Number of Adverse Events	Rate of adverse events (%)	Range of adverse events reported in individual studies (%)
Fever [20, 23, 27]	3	178	16	9.0	0–10.4
Irritability [23, 24]	2	28	10	35.7	7.7–60.0
Diarrhea [20, 23, 26, 27]	4	277	53	19.13	15.8–33.3
Hematochezia [26, 27]	2	243	8	3.3	1.4–6.3
Abdominal Distension [26, 27]	2	243	47	19.3	10.4–25.2
Vomiting [17, 20, 23, 26, 27]	5	480	78	16.25	0–40.0
Feeding Intolerance [24, 26]	2	109	17	15.6	0–16.7
Intussusception [27]	1	148	0	0	-
Volvulus [27]	1	148	0	0	-
Diaper Rash [23]	1	15	2	13.3	-

### Fever

Three studies reported data for the occurrence of fever (> 38.0 °C) after RV, including a total of 178 infants [20, 23, 27]. A total of 16 vaccinated infants from all three studies presented with fever after RV. Incidence rates reported were 0%, 7%, and 10%, respectively, with the highest number from a study reporting all incidences up to four weeks after the vaccination date.

### Gastrointestinal symptoms

Gastrointestinal symptoms were the most commonly reported adverse events after RV. For almost one fifth of infants, diarrhea (53/277, 19.1%) [20, 23, 26, 27] and abdominal distension (47/243, 19.3%) [26, 27] were observed, vomiting (78/480, 16.3%) [17, 20, 23, 26, 27] and feeding intolerance (17/109, 15.6%) [24, 26] were also common. One study qualitatively reported that more loose stool was observed in vaccinated infants, however, this was not deemed clinically relevant [17]. Hematochezia occurred in 8/243 (3.3%) infants [26, 27]. No cases of intussusception or volvulus were reported in one study investigating these outcomes [27]. No other study reported on the outcomes of intussusception or volvulus.

### Apnea-bradycardia episodes

A study conducted in the US in 2021 compared a cohort of premature infants (mean GA 27 6/7) to a historic control group of premature infants and compared events occurring within the study cohort a week prior and a week after receiving rotavirus vaccine with those occurring after the 2-months-vaccinations [17]. Although the study cohort exhibited a higher rate of apnea and/or bradycardia events, no significant difference was found between the post-vaccination period and the week preceding rotavirus vaccination when comparing the same individuals. Another study undertaken in Canada in 2015 reviewed medical notes for a period of four weeks after RV and found an increase in apnea-bradycardia episodes in 13/74 patients (18%) [27].

### Irritability

Irritability was reported by two smaller studies and occurred in 10/28 (35.7%) of vaccinated infants [23, 24]. The study that reported a high incidence of 60% (9/15) included only infants with pre-existing intestinal failure [23].

#### Shedding

Viral shedding was investigated in four studies [20, 22, 23, 29] with 229 patients. Time of investigation differed, and rates ranged from 26% to 100%. In those studies that repeatedly tested, shedding was higher after the first than after subsequent doses.

#### Nosocomial infection

Data for nosocomial infection was reported in four studies [20, 21, 26, 29]. Of those, three routinely collected stool samples from unvaccinated infants to monitor transmission [20, 21, 29]. Only one study set in a large US NICU found positive stool samples in 5 of 686 cases. Overall, less than 0.5% (5/1087) of unvaccinated infants had stool samples tested positive for the vaccine strain rotavirus. No study reported symptoms of acute gastroenteritis linked to positive stool samples in unvaccinated children (0/1888 patients).

#### Comparison with 2-month vaccination

In two studies, RV was compared to the 2-month vaccination without RV [17, 26]. One found episodes of apnea-bradycardia to occur more commonly after the two-month vaccination [17], in another, all cases of fever recorded were after co-administration of RV and the two-months-vaccination [26].

#### Infection precaution measurements

In a survey of UK neonatal units, 13/45 (28.8%) units stated taking infection precaution measurements, i.e., apron and gloves, for 48 h to one week after administration of RV [31]. The study did not report transmission rates. A large US cohort study that had low transmission rates (85/686 stool samples from unvaccinated children (0.7%) tested positive for RV5, no clinical signs for infection) reported no specific measures beyond the standard of care [29].

## Discussion

### Key findings

This review indicates that adverse events following rotavirus vaccination in premature infants are uncommon and generally mild. The available evidence on rotavirus vaccination in hospitalized premature infants is limited and of low quality, with included studies displaying substantial heterogeneity in design, methodology, and outcome reporting. Despite these limitations, the findings suggest that adverse events are infrequent and typically non-serious.

### Safety

Based on the findings of our review, no available evidence contradicts following national vaccination recommendations for preterm and medically ill infants at their chronological age.

Since premature infants are a particularly vulnerable population and have a significantly higher risk of severe rotavirus infection compared to full-term newborns, they could benefit particularly from the vaccine [12, 13]. In comparison to other vaccinations, the adverse event profile of RV for premature infants appears to be mild and should not be grounds for withholding the vaccination if the infant is clinically stable at the time of vaccination. While a 2021 US-study included in this review found apnea-bradycardia to increase after RV, it was not deemed clinically relevant [17]. A randomized controlled trial published in 2025 investigating apnea after the 2-month vaccination without RV including 224 infants reported a significant increase in clinically relevant apnea (i.e., escalated respiratory support or ventilation) [32].

Even though the data available on extremely premature infants is limited and only one study including 148 patients reported on the severe complication intussusception, it can be assumed that the relatively low side effect profile of the vaccination is offset by good protection against serious rotavirus infection. This also significantly reduces the risk of nosocomial rotavirus infection.

No studies report any infants with acute gastroenteritis due to a nosocomial transmission, and although the evidence stems from only three studies that regularly monitored stool samples for unvaccinated children, these studies include comparably large numbers of infants. We therefore conclude that the administration of RV in NICUs can be deemed safe. A study investigating the transmission after RV between twins in the home environment similarly found that although transmission was frequent, no cases of gastroenteritis occurred [33]. It has been suggested, but remains unclear, that this transmission of vaccine-strain rotavirus leads to the protection of unvaccinated infants [5].

For cases where discharge home is anticipated within the time window for the administration of the first dose, delaying the vaccination until the day of discharge may be an alternative to vaccination during the stay [34, 35]. However, this does not seem suitable as a general policy as it will still lead to some infants missing their vaccination cut-off.

#### Implications for clinical practice guidelines

Clinical practice guidelines are necessary to ensure consistent and, where possible, evidence-based care. They provide clear recommendations on monitoring, safety measures, and infection control, thereby reducing variability in clinical practice and minimizing preventable risks. In addition, standardized guidelines can support the harmonization of vaccination practices across institutions and countries, facilitating safer and more uniform implementation.

A review by Esposito et al. in 2018 concluded that increasing neonatologists’ knowledge on the efficacy and safety was an important step to achieving a higher vaccination rate [9]. While clinical practice guidelines provide an essential framework, their effectiveness depends on successful implementation at the local level. Measures to support this process may include structured educational approaches such as journal clubs to review current evidence and case-based discussions during clinical rounds to guide patient-specific decision-making. In addition, parental concerns about adding an extra burden during hospitalization may act as a barrier to timely vaccination, highlighting the importance of clear communication about the favorable safety profile and strong protection against severe rotavirus gastroenteritis to improve acceptance.

### Implications for further research

Further research should aim to address the substantial gaps in evidence by generating higher-quality and more standardized data on rotavirus vaccination in hospitalized premature infants. While intussusception is an important clinical concern, we found only one study with 148 infants explicitly reporting on this outcome [27]. In order to confidently recommend routine rotavirus vaccination, further studies should include this outcome. Another important evidence gap relates to infants with a history of abdominal surgery, such as those treated for necrotizing enterocolitis or intestinal perforation. Although some studies included infants with pre-existing gastrointestinal pathologies, no data are available for this high-risk subgroup specifically. Future research should therefore include predefined subgroup analyses to determine whether prior abdominal pathology or surgery modifies the risk profile of rotavirus vaccination.

Additionally, the existing evidence is insufficient to define an evidence-based monitoring interval. Future studies should therefore systematically assess the timing of potential adverse events following vaccination to inform clinically relevant post-vaccination monitoring recommendations.

To address these limitations, a prospective multicenter study with standardized protocols is warranted. Such a study should include clearly defined eligibility criteria (e.g., stratification by gestational age and comorbidities), systematic monitoring of adverse events over a predefined follow-up period (e.g., 42 days post-vaccination), and predefined subgroup analyses, including extremely premature infants and those with prior abdominal surgery. Systematic evaluation of viral shedding and transmission risk can clarify rate and clinical significance of nosocomial spreading, particularly in relation to hygiene practices and staffing models. While a randomized controlled trial may provide the highest level of evidence, large-scale prospective cohort studies may be more feasible in this vulnerable population while still allowing robust assessment of safety outcomes.

Furthermore, implementation-focused research investigating the above-mentioned strategies to foster vaccination uptake might help uncover specific barriers and identify beneficial factors. It should evaluate strategies to improve vaccine uptake, including standardized protocols, staff education, and parental counseling, in order to identify effective approaches for integrating rotavirus vaccination into routine NICU care.

## Conclusion

Rotavirus vaccination in hospitalized premature infants appears safe, yet significant knowledge gaps and inconsistent practices continue to limit its use in many NICUs. To ensure that this high-risk population receives timely protection, future efforts should focus on generating stronger evidence through standardized, prospective studies and on improving implementation strategies within clinical teams. Establishing clear protocols, enhancing provider education, and examining practical approaches to minimize missed vaccination opportunities will all be essential for translating current recommendations into reliable, equitable practice. 

## Supplementary Information

Supplementary Material 1.

## Data Availability

The datasets supporting the conclusions of this article are included within the article and its additional files.

## Abbreviations

NICU: Neonatal Intensive Care Unit
RV: Rotavirus Vaccination
