# Supplementary material for: In-hospital rotavirus vaccination in premature and medically ill infants: a systematic review of uptake and safety
Source: Mol Cell Pediatr. 2026 May 12;13:26. doi: 10.1186/s40348-026-00238-z (PMC13168386; doi:10.1186/s40348-026-00238-z)
Supplement: Supplementary file 1 — Supplementary Material 1. [file 40348_2026_238_MOESM1_ESM.pdf]

Appendix 1: Search Strategies

Pubmed
("Rotavirus Vaccines"[Mesh] OR "rotavirus vaccine*"[tiab] OR "rotavirus immunization"[tiab] OR "rotavirus immunisation"[tiab] OR "rotavirus vaccination"[tiab]) AND ("Infant, Newborn"[Mesh] OR neonat*[tiab] OR newborn*[tiab] OR "preterm"[tiab] OR "premature infant*"[tiab] OR "very low birth weight"[tiab] OR "low birth weight"[tiab]) AND ("Hospitals"[Mesh] OR "Intensive Care Units, Neonatal"[Mesh] OR "neonatal intensive care"[tiab] OR "NICU"[tiab] OR "inpatient"[tiab] OR "hospital setting"[tiab] OR "hospitalized"[tiab])

Embase
('rotavirus vaccine'/exp OR "rotavirus vaccine":ti,ab,kw) AND ('neonatal intensive care'/exp OR "neonatal intensive care":ti,ab,kw OR NICU:ti,ab,kw OR inpatient:ti,ab,kw OR "hospital ward":ti,ab,kw OR "in hospital":ti,ab,kw)
filter: „Medicine and Dentistry“ and „Research Articles“

Web of Science
(rotavirus OR rotavirus vaccine OR rotavirus vaccination OR rotavirus immunization OR rotavirus prevention) AND (hospitalized newborns OR hospitalized infants OR hospitalized neonates OR premature newborns OR premature infants OR premature neonates OR preterm newborns OR preterm infants OR preterm neonates) AND (nicu OR neonatal intensive care unit OR inpatient setting OR hospital setting) AND (safety OR efficacy OR effectiveness OR implementation OR feasibility OR outcomes OR adverse effects OR complications)
